# Life on Human Surfaces: Skin Metagenomics

**DOI:** 10.1371/journal.pone.0065288

**Published:** 2013-06-12

**Authors:** Alban Mathieu, Tom O. Delmont, Timothy M. Vogel, Patrick Robe, Renaud Nalin, Pascal Simonet

**Affiliations:** 1 Environmental Microbial Genomics group, Laboratoire Ampere, Ecole Centrale de Lyon, Université de Lyon, Ecully, France; 2 LibraGen, Toulouse, France; Charité-University Medicine Berlin, Germany

## Abstract

The human skin microbiome could provide another example, after the gut, of the strong positive or negative impact that human colonizing bacteria can have on health. Deciphering functional diversity and dynamics within human skin microbial communities is critical for understanding their involvement and for developing the appropriate substances for improving or correcting their action. We present a direct PCR-free high throughput sequencing approach to unravel the human skin microbiota specificities through metagenomic dataset analysis and inter-environmental comparison. The approach provided access to the functions carried out by dominant skin colonizing taxa, including *Corynebacterium*, *Staphylococcus* and *Propionibacterium*, revealing their specific capabilities to interact with and exploit compounds from the human skin. These functions, which clearly illustrate the unique life style of the skin microbial communities, stand as invaluable investigation targets for understanding and potentially modifying bacterial interactions with the human host with the objective of increasing health and well being.

## Introduction

The mutualistic interactions between humans and microbial communities that result from long co-evolutionary processes involve the skin separating and protecting the human body against its outside environment. Selected by both environmental and human characteristics, skin microorganisms are not qualitatively and quantitatively evenly distributed on the body, since they range from less than 10^2^ to more than 10^7^ cells.cm^−2^ as a function of the colonized site [1]. A widely used approach for studying microbial DNA extracted from skin is through its *in vitro* enzymatic amplification by targeting the *rrs* gene with “universal” primers [2]. This technique, not constrained by the low amounts of microbial DNA extractible from skin, led to the description of the skin microbiota as much more diverse than previously estimated by cultivation-based methods, with strong spatial variations as well as consistent individual host specificities [3], [4], [5], [6], [7]. However, a better understanding of the skin microbiota’s role in protecting against pathogens and in improving human health requires investigation beyond the taxonomic inventory of bacteria. Specific activities associated with functional genes need to be characterized. This can be achieved by sequence-based skin microbiome explorations via high throughput shotgun sequencing of metagenomic DNA or by associating species to function with previously sequenced genomes. Due to differences in function even within the same microbial species [8], we decided to build shotgun metagenomic libraries for the skin microbiome despite the requirement for high DNA concentrations.

## Materials and Methods

The experimental design performed here was to neglect taxonomic variations over time and between body locations and to consider the skin microbiota as a complete ecosystem sharing the ability to develop on skin whoever the host and whatever the environmental conditions. Thus, the bottleneck of DNA recovery and PCR bias was overcame by sequentially sampling individuals on different parts of their body over time until the pooled extracted DNA satisfied sequencing requirements.

Two 25–26 year old Caucasian males without any symptoms of skin disease were subjected to sampling of different parts of their bodies, including palms, face, axilla, feet and retro auricular crease, every two days for a week. Because of the non-invasive approach to sample, the ethic statement was accepted by the commission CPP of SUD EST IV Centre Léon Bérard (Personnal protection commission from the Centre Léon Bérard). Human subjects remain anonymous and the informed consent was written. For each individual, environmental (skin surface) DNA samples were extracted and purified according to the protocol described by Griffiths [9] and subsequently pooled. The same sampling effort was repeated for a second week in order to constitute a second replicate of the global human skin microbiota for each of the two individuals tested (detailed protocol accessible in Protocol S1). These four DNA samples (>3 micrograms each) were tagged separately and sequenced (Titanium pyrosequencing technology, reads size average of 184±138 bp). Artificial duplicates were deleted using cd-hit-454 with default parameters [10] and human genome related reads were deleted prior to data analysis. Human DNA contamination was low in both datasets: individual 1 (0.53% ±0.3%) and individual 2 (2.89% ±0.7%).

### Generating and Annotating Metagenomic Datasets

Sequence reads were compared to reference databases in order to annotate and classify them in functional subsystems: the generated metagenomic datasets (read average number = 33657±4835) were uploaded on the MG RAST v3 public server [11] for both taxonomic (Lowest common ancestor approach [12]) and functional annotations (e-value cut-off of 10^−5^). The relative distribution of these sequences was normalized as a function of the total number of annotated reads in each dataset [13]. Skin metagenome datasets were compared to 65 publicly available metagenomic datasets (MG-RAST, accession numbers in table S3) from different environments, such as soil, oceans, hot springs, acid mine drainage, sludge wastewater treatment plant, air, deep ocean, human gut, cow, mouse and chicken gut.

Using the SEED database (>2000 reference genomes [14]), 49% (±14%) of the generated sequences from human skin microbiota were associated to a unique genus. Genomes related to *Corynebacterium*, *Staphylococcus* and *Propionibacterium* dominated these metagenomic datasets (table S1) and provided information to connect taxa and functions.

## Results and Discussion

### Specificity of the Human Skin Microbiota

**Figure 1:**
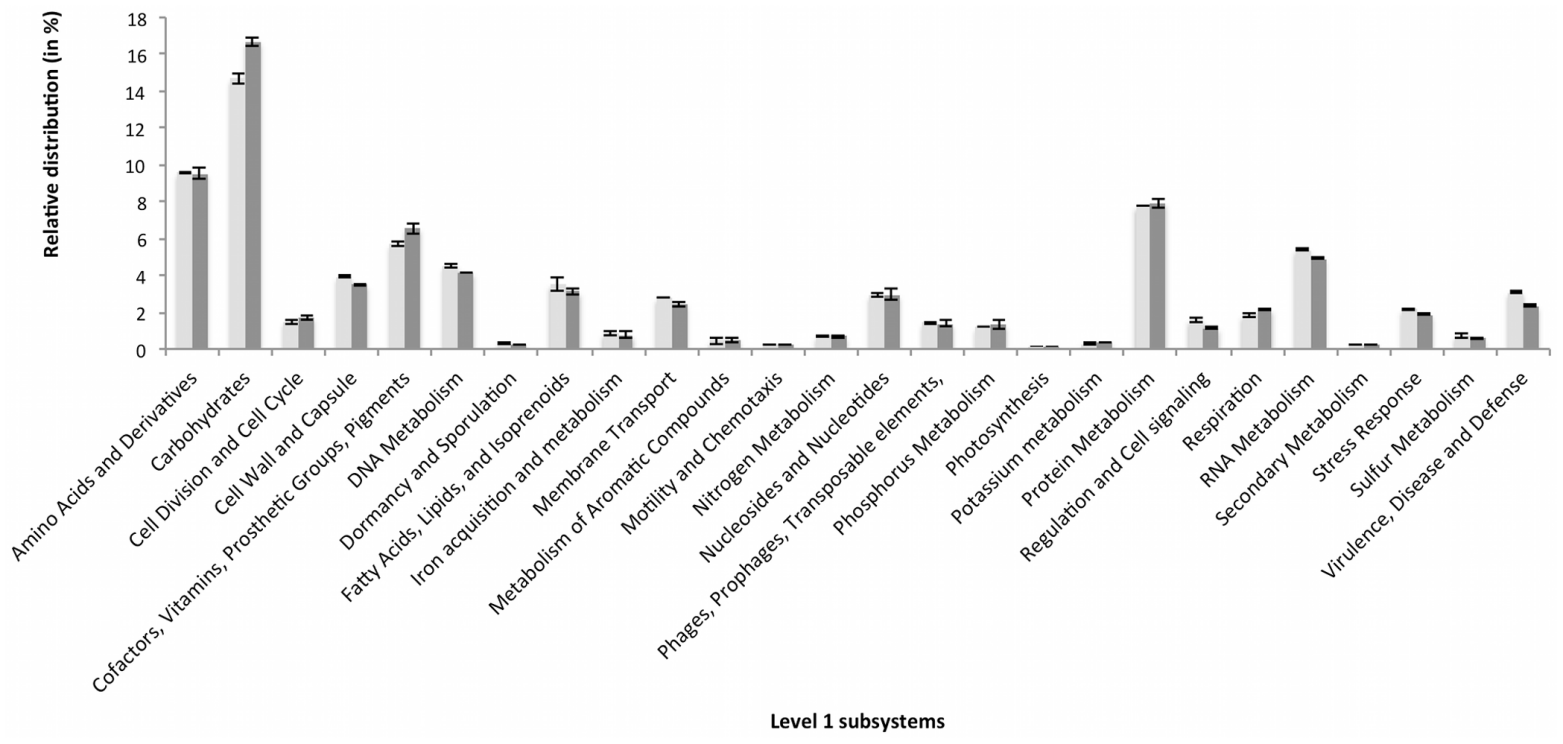
Functional distribution of skin metagenomes. Relative distribution of skin metagenome reads assigned to the 26 general SEED functional subsystems (e-value cut-off: 10^−5^) expressed as a percentage of all annotated reads from the four skin datasets used in this study: 2 from the individual 1 (light grey); 2 from the individual 2 (dark grey). Each functional distribution was compared and no significant difference is observed between the two individuals using Welch’s test (p-value <0.05).

**Figure 2:**
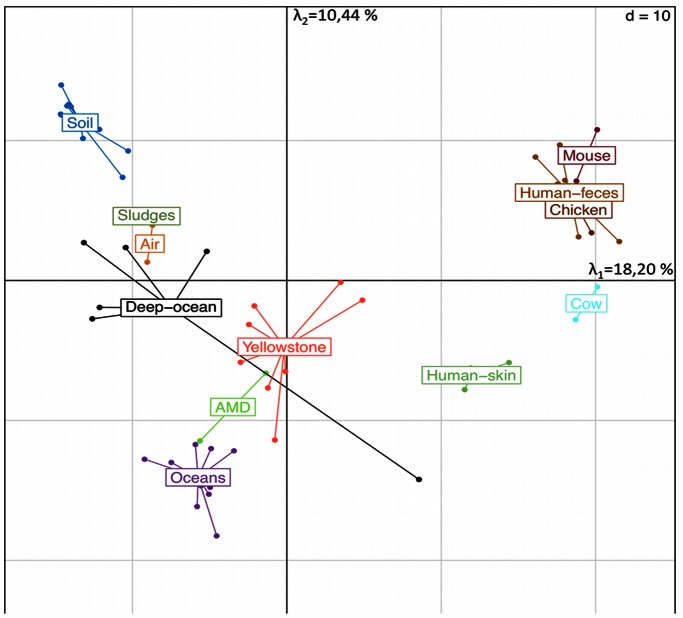
Inter-environmental comparison with a principal component analysis. Comparison of the four skin datasets generated with 65 other publicly available environmental metagenomic datasets by PCA using the relative distribution of annotated reads in each dataset. In this case, data were obtained using the same cut-off (e-value cut-off <10^−5^) and SEED functional level 3 subsystem (deeper characterization, ≈ 800 functions are compared in each dataset).

Using the SEED annotation, 72.2% (±2,2%) of the 22 456 (±2765) predicted proteins from human skin microbiota were functionally annotated. Most of the sequences were related to a limited number of general subsystems (fig.1) like carbohydrates, amino acids and derivatives, as well as protein metabolism. In contrast, other general subsystems are less represented (*e.g*., motility and chemotaxis). Homogeneity of the functional distributions among the different datasets can be explained by the functions observed at the general subsystem level (*e.g.* photosynthesis, respiration…). Specifically, hundreds of subsystems can be simultaneously compared when studying datasets at more detailed functional levels. However it was not possible to explain the functional specificity of the skin microbiome without comparing it to other environments. Thus, principal component analysis (PCA) based on the relative functional distribution of the reads from 69 datasets (annotated by SEED functional level 3 subsystem, 800 functions compared), including human skin and feces, revealed an apparent specificity of skin microbial communities since their related datasets clustered separate from those generated on the 12 other investigated environments (fig. 2). While methodological differences could induce biases among the different metagenomic datasets, these do not compromise their comparison as has been demonstrated previously [13]. Among the 626 functional subsystems detected in human skin related datasets, 52 possessed a distribution significantly different from the 65 other datasets (table S2). These 52 functional subsystems were reviewed to decipher the specific nature of the skin core-(meta)genome and its potential contribution to human health. In order to best represent the skin microbiome’s lifestyle, six of these were analyzed in more detail. Other functions are accessible in the table S2.

### Specific Functional Distributions

**Figure 3:**
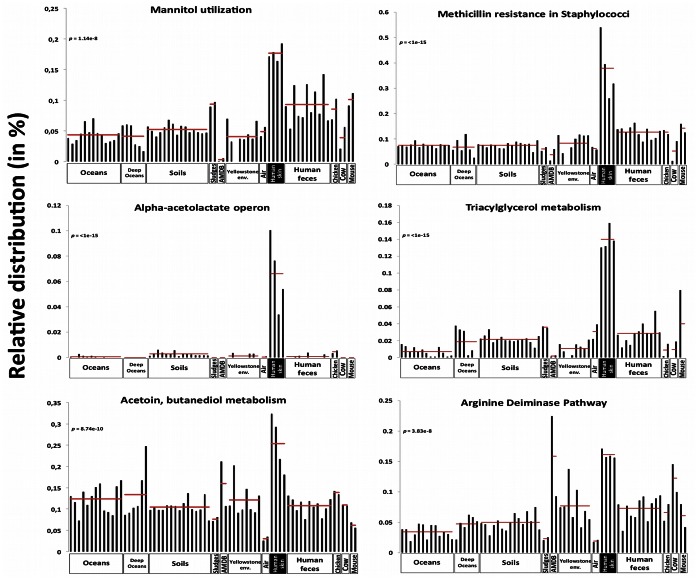
Representative functions of the skin microbiome lifestyle among the 69 metagenomic datasets. Relative distribution of reads assigned to 6 functions of the SEED level 3 functional subsystems among 69 metagenomes (based on MG-RAST v3 annotation, e-value cut-off <10^−5^) and having a different distribution in the skin datasets compared to the 65 other datasets. Data are normalized by the total annotated sequences and are expressed in percentage. Each histogram represents the relative distribution of a unique metagenome, horizontal lines represent the mean of the relative distribution in each 12 environments (oceans, deep oceans, soils, wastewater treatment sludges, acid mine drainage biofilm, hot springs, air, human skin, human feces, chicken gut, cow gut and mouse gut). P-value is the probabilty that the difference observed between skin and other environments is insignificant (Whelch’s test, other environments are grouped as one for the statistical test and each function is compared in each dataset).

One of the major advantages of metagenomic approaches is the possibility of describing a complex community without any *a priori* views concerning taxa and/or functions prior to exploiting sequence data. The human skin surface microbial communities potentially possess strong capacities for interacting with their environment based on inter-ecosystem comparisons (fig. 3). The functional potential of skin bacteria indicates that these bacteria are strongly adapted to the exploitation of compounds produced by the human skin, including sugars, lipids (and iron), and petrobactin-mediated iron uptake systems were detected in the metagenome datasets. The presence of lactic acid generated by sweat (>99 µg/ml) [15] and of a sugar, such as sialic acid, common to humans and animals justifies the predominance of respective catabolic genes in our skin metagenome datasets. Similarly, the detection of a high proportion of sequences related to triacylglycerol catabolism, a skin lipid critically involved in epidermal permeability, suggests its utilization as a carbon source and triacylglycerol catabolism could also provide a significant contribution to the lipases produced by the skin for preventing lipid accumulation as in the ichthyosis pathology [16].

The role of the microbiota in regulating another critical healthy state parameter (skin acidity), which controls the permeability barrier homeostasis, is also highlighted by numerous functional subsystems associated with acid resistance detected in the databases. For instance, acidification ecosystem preservation could explain the bacterial adaptive strategy of using the butanediolic fermentation as deduced from detection of alpha acetolactate and acetoin butanediol metabolism genes for transforming pyruvate into the final product (2,3-butanediol) rather than a mixed acid fermentation. The predominance of genes involved in the arginine deiminase metabolism [17] in the metagenome datasets confirms the tolerance of bacteria to skin acidity [15]. The skin metagenome analysis also brings new clues about the extensive spread of antibiotic resistance genes among bacteria. Within the human skin *Staphylococcus* populations of the two individuals, various *Staphylococci* seem to be intrinsically resistant to methicillin (fig. 3), although neither of the two individuals had recent contact with a methicillin-rich environment (hospitalization and/or methicillin treatment). Moreover, the level of teicoplanin and bacitracin resistance genes was particularly high in the sequence datasets.

Finally, metagenomic analysis also contributes to the raising new and unexpected questions regarding bacteria adaptation and interactions with the human host. For example, mannitol catabolic pathway sequences were detected at an unexpected high level (fig. 3) even though this common sugar, usually transformed into fructose derivates before entering the glycolysis, was not referenced as a human skin component.

## Supporting Information

Table S1 **Most abundant genera in the skin metagenomic datasets.** Relative distribution of the 16 most detected genera in the skin metagenomes using the lowest common ancestor approach. Data were normalized by the total annotated sequences.+symbolize the detection in *rrs*-based studies, - the absence; and then the reference.
(DOCX)

Table S2 **52 functions statistically up-represented on the skin metagenomic datasets.** Functions of the SEED level 3 subsystem differentially detected in skin datasets in comparison to other datasets (Whelch’s test, other environments are grouped as one for the statistical test and each function is compared in each dataset).
(DOCX)

Table S3 **Accession number of metagenomic datasets (MG-RAST v3).** Accession number of metagenomes: These accession numbers correspond to metagenomes available on MG-RASTv3 server (http://metagenomics. anl.gov/).
(DOCX)

Protocol S1 **Sampling, DNA extraction.**
(DOC)
