# Supplementary material for: Life on Human Surfaces: Skin Metagenomics
Source: PLoS One. 2013 Jun 12;8(6):e65288. doi: 10.1371/journal.pone.0065288 (PMC3680502; doi:10.1371/journal.pone.0065288)
Supplement: Table S1 — Most abundant genera in the skin metagenomic datasets. Relative distribution of the 16 most detected genera in the skin metagenomes using the lowest common ancestor approach. Data were normalized by the total annotated sequences.+symbolize the detection in rrs-based studies, - the absence; and then the reference. (DOCX) [file pone.0065288.s001.docx]

**Table S1: Most abundant genera on the skin metagenomic datasets**

Relative distribution of the 16 most detected genera in the skin metagenomes using the Lowest common ancestor approach. Data were normalized with the total of annotated sequences. + symbolize the detection in rrs- based studies, - the absence ; then the publication associated.

| genus | Relative frequency in individual n°1 (%) | Relative frequency in individual n°2 (%) | Detection in *rrs*-based studies | |
| --- | --- | --- | --- | --- |
| Corynebacterium | 38,98 ±1,69 | 31,83 ±4,85 | + | ^[3]^ |
| Propionibacterium | 7,92 ±0,98 | 38,37 ±5,05 | + | ^[3]^ |
| Staphylococcus | 33,3 ±3,66 | 13,42 ±0,98 | + | ^[3]^ |
| Brevibacterium | 0,57 ±0,50 | 0,1 ±0,06 | + | ^[6]^ |
| Rothia | 0,43 ±0,01 | 0,06 ±0,00 | + | ^[6]^ |
| Malassezia | 0,28 ±0,08 | 0,41 ±0,14 | + Eukaryote |  |
| Cupriavidus | 0,36 ±0,36 | 0 ±0,00 | - |  |
| Streptococcus | 0,32 ±0,19 | 0,2 ±0,05 | + | ^[3]^ |
| Anaerococcus | 0,2 ±0,04 | 0,08 ±0,02 | + | ^[3]^ |
| Micrococcus | 0,2 ±0,08 | 0,08 ±0,02 | + | ^[3]^ |
| Kocuria | 0,17 ±0,02 | 0,06 ±0,01 | + | ^[6]^ |
| Cyanothece | 0,02 ±0,02 | 0,17 ±0,04 | - |  |
| Eremococcus | 0,14 ±0,00 | 0 ±0,00 | + | ^[6]^ |
| Coprobacillus | 0,03 ±0,01 | 0,14 ±0,03 | - |  |
| Arthrobacter | 0,13 ±0,07 | 0,03 ±0,02 | - |  |
| Actinomyces | 0,1 ±0,01 | 0,07 ±0,01 | + | ^[3]^ |

Bibliography:

3. Costello, E. K. *et al.* Bacterial Community Variation in Human Body Habitats Across Space and Time. *Science* **326**, 1694 –1697 (2009).

6. Gao, Z., Tseng, C., Pei, Z. & Blaser, M. J. Molecular analysis of human forearm superficial skin bacterial biota. *Proceedings of the National Academy of Sciences* **104**, 2927 –2932 (2007).
