# Supplementary material for: Life on Human Surfaces: Skin Metagenomics
Source: PLoS One. 2013 Jun 12;8(6):e65288. doi: 10.1371/journal.pone.0065288 (PMC3680502; doi:10.1371/journal.pone.0065288)
Supplement: Table S2 — 52 functions statistically up-represented on the skin metagenomic datasets. Functions of the SEED level 3 subsystem differentially detected in skin datasets in comparison to other datasets (Whelch’s test, other environments are grouped as one for the statistical test and each function is compared in each dataset). (DOCX) [file pone.0065288.s002.docx]

**Table S2: 52 functions statistically up-represented on the skin metagenomic datasets**

Functions of MG-RAST level 3 subsystem differentially detected in skin datasets in comparison to other environments datasets (Whelch’s test, other environments are grouped as one unique environment for the statistical test).

| MG-RAST level 3 subsystem | P-value | Skin: mean relative frequency ± standard deviation (%) | Other environments: mean relative frequency ± standard deviation (%) |
| --- | --- | --- | --- |
| Alpha-acetolactate operon | 4,36E-30 | 0,08 ±0,03 | 00 |
| Cannabinoid biosynthesis | 3,77E-25 | 0,05 ±0,01 | 0,010 |
| Triacylglycerol metabolism | 1,70E-24 | 0,18 ±0,02 | 0,02 ±0,02 |
| Glutathione analogs: mycothiol | 2,80E-24 | 0,2 ±0,05 | 0,01 ±0,02 |
| Multi-subunit cation antiporter | 3,14E-24 | 0,42 ±0,15 | 0,02 ±0,03 |
| Biofilm formation in Staphylococcus | 1,46E-22 | 0,23 ±0,12 | 0 ±0,01 |
| Adhesins in Staphylococcus | 2,41E-22 | 0,3 ±0,13 | 0,01 ±0,02 |
| Petrobactin-mediated iron uptake system | 9,86E-22 | 0,05 ±0,03 | 00 |
| Methicillin resistance in Staphylococci | 2,19E-20 | 0,48 ±0,13 | 0,11 ±0,04 |
| D-Alanyl Lipoteichoic Acid Biosynthesis | 5,18E-18 | 0,04 ±0,02 | 00 |
| Lactate utilization | 2,57E-15 | 0,26 ±0,02 | 0,07 ±0,04 |
| Bacitracin Stress Response | 1,50E-14 | 0,05 ±0,03 | 0 ±0,01 |
| Cytolysin and Lipase operon in Vibrio | 1,52E-14 | 0,03 ±0,02 | 00 |
| Toxin-Antitoxin MT1 | 1,20E-13 | 0,01 ±0,01 | 00 |
| Anaerobic Oxidative Degradation of L-Ornithine | 2,17E-13 | 0,09 ±0,07 | 0 ±0,01 |
| SecY2-SecA2 Specialized Transport System | 3,15E-13 | 0,08 ±0,03 | 0 ±0,01 |
| Teicoplanin-resistance in Staphylococcus | 1,95E-12 | 0,02 ±0,02 | 00 |
| Siderophore Anthrachelin | 3,76E-12 | 0,03 ±0,02 | 00 |
| CoA disulfide thiol-disulfide redox system | 1,38E-11 | 0,02 ±0,01 | 00 |
| Mannitol Utilization | 3,32E-11 | 0,23 ±0,01 | 0,07 ±0,04 |
| Fructose utilization | 8,73E-11 | 0,33 ±0,05 | 0,07 ±0,07 |
| Prophage lysogenic conversion modules | 1,38E-10 | 0,03 ±0,01 | 0 ±0,01 |
| Acetoin, butanediol metabolism | 2,23E-10 | 0,32 ±0,07 | 0,14 ±0,04 |
| Teichoic and lipoteichoic acids biosynthesis | 2,94E-10 | 0,31 ±0,1 | 0,08 ±0,06 |
| Pyruvate Alanine Serine Interconversions | 1,03E-09 | 0,37 ±0,04 | 0,19 ±0,05 |
| Glycine and Serine Utilization | 2,20E-09 | 0,87 ±0,05 | 0,54 ±0,09 |
| CBSS-196164.1.peg.461 | 2,20E-09 | 0,19 ±0,02 | 0,06 ±0,04 |
| Menaquinone and Phylloquinone Biosynthesis | 3,08E-09 | 0,2 ±0,03 | 0,08 ±0,03 |
| Dihydroxyacetone kinases | 8,51E-09 | 0,08 ±0,04 | 0,02 ±0,02 |
| Staphylococcal pathogenicity islands SaPI | 1,10E-08 | 0,49 ±0,08 | 0,24 ±0,07 |
| Murein hydrolase regulation and cell death | 1,11E-08 | 0,11 ±0,06 | 0,03 ±0,02 |
| Purine conversions | 2,85E-08 | 1,02 ±0,06 | 0,67 ±0,11 |
| Glycerol and Glycerol-3-phosphate Uptake and Utilization | 3,12E-08 | 0,36 ±0,02 | 0,2 ±0,05 |
| Arginine Deiminase Pathway | 3,83E-08 | 0,21 ±0,01 | 0,07 ±0,04 |
| Sialic Acid Metabolism | 8,69E-08 | 0,86 ±0,08 | 0,41 ±0,15 |
| L-Cystine Uptake and Metabolism | 1,23E-07 | 0,08 ±0,03 | 0,03 ±0,02 |
| Galactose-inducible PTS | 1,48E-07 | 0,1 ±0,01 | 0,04 ±0,02 |
| TCA Cycle | 2,01E-07 | 1,47 ±0,16 | 0,73 ±0,25 |
| DNA repair, bacterial RecBCD pathway | 4,95E-07 | 0,2 ±0,07 | 0,07 ±0,04 |
| Cluster-based Subsystem Grouping Hypotheticals - perhaps Proteosome Related | 5,32E-07 | 0,21 ±0,07 | 0,06 ±0,05 |
| Alpha-Amylase locus in Streptocococcus | 1,45E-06 | 0,06 ±0,01 | 0,02 ±0,01 |
| D-gluconate and ketogluconates metabolism | 2,03E-06 | 0,27 ±0,07 | 0,11 ±0,06 |
| At5g63420 | 2,14E-06 | 0,59 ±0,09 | 0,36 ±0,09 |
| D-Sorbitol(D-Glucitol) and L-Sorbose Utilization | 2,27E-06 | 0,04 ±0,03 | 0,01 ±0,01 |
| Proton-dependent Peptide Transporters | 2,45E-06 | 0,07 ±0,02 | 0,03 ±0,02 |
| Acetone Butanol Ethanol Synthesis | 2,61E-06 | 0,17 ±0,01 | 0,4 ±0,09 |
| CBSS-262719.3.peg.410 | 3,10E-06 | 0,19 ±0,07 | 0,1 ±0,03 |
| Ubiquinone Menaquinone-cytochrome c reductase complexes | 3,56E-06 | 0,19 ±0,02 | 0,06 ±0,05 |
| KDO2-Lipid A biosynthesis | 5,39E-06 | 0,01 ±0,01 | 0,27 ±0,1 |
| CBSS-280355.3.peg.2835 | 6,09E-06 | 0,28 ±0,04 | 0,18 ±0,04 |
| tRNA aminoacylation, Lys | 7,10E-06 | 0,15 ±0,05 | 0,08 ±0,03 |
| Vibrio Core Oligosaccharide Biosynthesis | 7,71E-06 | 0,010 | 0,09 ±0,03 |
