# Supplementary material for: Life on Human Surfaces: Skin Metagenomics
Source: PLoS One. 2013 Jun 12;8(6):e65288. doi: 10.1371/journal.pone.0065288 (PMC3680502; doi:10.1371/journal.pone.0065288)
Supplement: Table S3 — Accession number of metagenomic datasets (MG-RAST v3). Accession number of metagenomes: These accession numbers correspond to metagenomes available on MG-RASTv3 server (http://metagenomics. anl.gov/). (DOCX) [file pone.0065288.s003.docx]

**Table S3**: Accession number of metagenomic datasets (MG-RAST v3)

Accession number of metagenomes: These accession numbers correspond to metagenomes available on MG-RASTv3 server (http://metagenomics. anl.gov/).

| *Oceans* | | | | | | |
| --- | --- | --- | --- | --- | --- | --- |
| 4441573.3 | 4441574.3 | 4441576.3 | 4441577.3 | 4441591.3 | 4441660.3 | 4443716.3 |
| 4443697.3 | 4443713.3 | 4443714.3 | 4443725.3 | 4443729.3 | 4443688.3 |  |
| *Deep oceans* | | | | | | |
| 4441619.3 | 4441656.4 | 4441620.3 | 4441663.3 | 4442503.3 | 4442500.4 |  |
| *Soils* | | | | | | |
| 4441091.3 | 4446153.3 |  | | | | |
| http://metasoil.univ-lyon1.fr for metagenomes corresponding to Rothamsted Park Grass soil experiment. | | | | | | |
| *Phosphorus removing sludges* | | | | | | |
| 4441092.3 | 4441093.3 |  | | | | |
| *Acid Mine Drainage Biofilms* | | | | | | |
| 4441137.3 | 4441138.3 |  | | | | |
| *Singapore indoor polluted airs* | | | | | | |
| 4447940.3 | 4447941.3 |  | | | | |
| *Human feces* | | | | | | |
| 4447940.3 | 4447941.3 | 4440616.3 | 4440611.3 | 4440939.3 | 4440943.3 | 4440452.7. |
| 4440825.3 | 4440595.4 | 4440460.5 | 4440942.3 | 4440614.3 | 4440613.3 |  |
| *Chicken Cecum* | | | | | | |
| 4440283.3 | 4440284.3 |  | | | | |
| *Mouse cecum* | | | | | | |
| 4440463.3 | 4440464.3 |  | | | | |
| *Cow rumen* | | | | | | |
| 4441679.3 | 4441680.3 |  | | | | |
| *Yellowstone* | | | | | | |
| 4453438.3 | 4453437.3 | 4453439.3 | 4453440.3 | 4453441.3 | 4453513.3 | 4453442.3 |
| 453443.3 |  | | | | | |
| *Skin* | | | | | | |
| http://www.genomenviron.org/Projects/skinmicrobiome.html for metagenomes corresponding to the human skin experiment. | | | | | | |
