# Supplementary material for: Life on Human Surfaces: Skin Metagenomics
Source: PLoS One. 2013 Jun 12;8(6):e65288. doi: 10.1371/journal.pone.0065288 (PMC3680502; doi:10.1371/journal.pone.0065288)
Supplement: Protocol S1 — Sampling, DNA extraction. (DOC) [file pone.0065288.s004.doc]

**Supporting information**

**Protocol S1:**

Sampling, DNA extraction:

Individuals were swabbed every two days during March 28th and April 15th 2011 on the palm surfaces, face (including nose and forehead), axilla, feet and retroauricular crease. Skin samples were taken at the end of the day and subjects provided characteristic on their hygiene and skin conditions. Sterile gazes are pre-moistened with a sterile solution of 0,15M NaCl– 0,1% Tween 20 and one gaze was used for each part of the body. Gazes samples were collected in a 50 ml Falcon® tube for each day of extraction. Each tube was fully filled on with Nacl-Tween 20 solution, horizontally shacked 15 min at 800 rpm, then sonicated 5 min at 44-48 kHz (ULTRASonik, 28H). The NaCl-Tween 20 solution was transferred in new tubes and gazes spined-dry under sterile condition to collect the biggest volume of the solution. Samples were then centrifuged at 8000 rpm during 30 min to obtain a cell pellet. DNA extraction was performed based on a physical lysis^8^. Briefly the cell pellet was resuspended with a hexadecyltrimethylammonium bromide (CTAB) extraction buffer (0,5 ml) and phenol-chloroform-isoamyl alcohol 25:24:1 (0,5ml). Cells were lysed 30sec at 5,5 m/s using the FastPrep FP120 bead beating system (Bio-101, Vista, California). Samples were centrifuged 10 min, 4°C at 11 000 rpm. The aqueous phase was collected and the precipitation done with 2 volumes of ethanol 100% and 1/10 volume of NaCl 5M at 4°C overnight. Then purification was performed using the Illustra GFX purification kit (GE HealthCare, Pittsburgh, USA) to obtain a high purity of DNA. Two pools of DNA were kinetically collected for the two individuals. Four to six extractions for each DNA pool were cumulated to reach the 3 µg of DNA required to generate a metagenome with the actual pyrosequencing titanium sequencing technology.

Sequencing and annotation:

Sequencing was performed with the 454 technology, 1/6e of an entire run was required to obtain 33657 ± 4835 reads for each tag (1 tag for 1 sample, 120 000 - 130 000 in total). Sequences were uploaded on the MG-RAST online server for the sequence annotation (<http://metagenomics.nmpdr.org/>). The strategy of the lowest common ancestor was used for the taxonomic annotation. Only hits classified in a unique genus could be taking into account, reads having not discriminants were classified in an upper taxonomic level. Functional annotation was made with the SEED subsystem annotation. Only hits with an e-value<10^-5^ were considered to be significant for further analyses. The taxa/functional distributions were exported to the STAMP software for statistical analyses^17^. Welch’s statistical test was applied to compare groups 2 by 2, the skin datasets versus the other environmental datasets (Soil; oceans; Yellowstone; acid mine drainage; sludge wastewater treatment plant; air; deep ocean; human gut; cow, mouse and chicken gut; accession number in table S3).
